# Reforming medical education admission and training in low- and middle-income countries: who gets admitted and why it matters

**DOI:** 10.1186/s12960-019-0426-9

**Published:** 2019-12-02

**Authors:** Katherine Tumlinson, Dilshad Jaff, Barbara Stilwell, Dickens Otieno Onyango, Kenneth L. Leonard

**Affiliations:** 1 Department of Maternal and Child Health, Gillings School of Global Public Health, The University of North Carolina at Chapel Hill, 135 Dauer Drive, 405a Rosenau Hall, CB #7445, Chapel Hill, NC 27599 USA; 2 Carolina Population Center at The University of North Carolina at Chapel Hill, Chapel Hill, NC USA; 3 Nursing Now, London, UK; 4 Kisumu County Health Department, Kisumu, Kenya; 5 College of Agriculture and Natural Resources, University of Maryland, College Park, MD USA

**Keywords:** Quality of care, Healthcare provider performance, Healthcare provider motivation, Health system reform, Low- and middle-income countries

## Abstract

Recent studies reveal public-sector healthcare providers in low- and middle-income countries (LMICs) are frequently absent from work, solicit informal payments for service delivery, and engage in disrespectful or abusive treatment of patients. While extrinsic factors may foster and facilitate these negative practices, it is not often feasible to alter the external environment in low-resource settings. In contrast, healthcare professionals with strong intrinsic motivation and a desire to serve the needs of their community are less likely to engage in these negative behaviors and may draw upon internal incentives to deliver a high quality of care. Reforming medical education admission and training practices in LMICs is one promising strategy for increasing the prevalence of medical professionals with strong intrinsic motivation.

## Introduction

A primary public health goal in low- and middle-income countries (LMICs) is improved quality of healthcare. High-quality healthcare services result in increased service utilization and better health outcomes and are heavily dependent on the performance of individual healthcare providers [1]. In recent years, however, some healthcare providers^1^ in LMICs have been found to engage in behaviors that prioritize personal profit and desires over high-quality service delivery. For example, recent studies reveal healthcare providers within public facilities are frequently absent from work, solicit informal payment for service delivery, and engage in disrespectful or abusive treatment of patients [2–5]. These negative provider behaviors are prevalent in many LMICs [3] and are hypothesized to be especially prevalent in rural and remote facilities where supervisory visits are infrequent. Improving workforce efficiency is important in all country contexts but is particularly important in LMICs where disease burden is highest and workforce shortages are greatest. The objective of this commentary is to describe promising, feasible, and sustainable recommendations for correcting negative provider behaviors; our recommendations are designed to address the systemic challenge of low intrinsic motivation among providers in developing countries.

## Negative provider behaviors: impact and motivational factors

The systemic nature of negative provider behaviors in LMICs contributes to poor quality of care which, in turn, likely contributes to low service utilization and poor health outcomes. Additionally, as patients become increasingly dissatisfied with, and distrustful of, the medical community, providers in some countries are experiencing verbal harassment, physical attack, and death threats [6]. As such, failure to identify and address negative provider behavior could have wide-reaching consequences for both patients and health professionals. Finding feasible and sustainable means of redressing negative provider behaviors, therefore, warrants greater attention.

Identifying promising solutions necessitates consideration of the many potential factors that impact provider motivation. Factors that impact work motivation are both internal to the worker—such as self-concept, or expectation of reward or punishment—and external to the worker such as environment or equipment [7]. Below we discuss factors that we hypothesize impact provider motivations in health settings in LMICs, informed by personal observation, first-hand experience, and review of previous studies. We consider the role of both extrinsic and intrinsic motivating factors. We also consider how extrinsic and intrinsic factors differ as points of feasible and sustainable intervention and offer our recommendations for reform.

### Extrinsic motivational factors

Extrinsic motivational factors are those factors that are largely outside of the individual clinician’s control, but which effect their work environment or job satisfaction. Most people are impacted at least to a small degree by extrinsic factors but those who are highly driven by external rewards and personal profit may be more susceptible to engaging in harmful practices while employed in suboptimal work environments. Extrinsic factors impact provider behavior and are often embedded in the larger health system of LMICs. Intervening to change or improve the extrinsic factors impacting provider behavior (such as increased supervision, better infrastructure, or higher wages) can be costly and therefore may be challenging or unfeasible in resource-constrained areas [8].

Examples of extrinsic factors impacting provider behavior:

- Infrequent or inadequate supervision with poor accountability. Providers in many LMICs do not have routine and supportive supervision [9]. Remote facilities may also be staffed by a single provider, eliminating the possibility that a colleague may impose accountability. Additionally, public-sector providers are unlikely to be rewarded for good behavior or punished for bad behavior, providing little external motivation to excel within the workplace.
- Poor facility infrastructure. Unreliable electricity, lack of running water, and absence of critical supplies, equipment, and commodities all negatively impact the quality of service delivery. Additionally, poor infrastructure can make it difficult to maintain high performance standards when faced with the reality of not having the necessary tools or resources to deliver services.
- Inadequate compensation. Providers in many LMICs complain that their wages have been static for long periods of time and have failed to keep pace with the rising cost of living. Many also complain that their wages do not honor or reflect the high degree of skill and effort required for quality services [5]. Additionally, healthcare providers who do not receive scheduled promotions (or who complete additional studies and are not promoted to reflect that effort) are likely to be demoralized. Further, perceptions around the adequacy of wages may be impacted by societal expectations. In many LMICs, there is a societal belief that healthcare providers are wealthy, yet public-sector wages may be modest. To meet this expectation of prosperity, healthcare providers may top up public-sector wages by neglecting their government post to pursue simultaneous wages in the private sector.
- Compromised personal safety. Some healthcare providers may have to contend with a lack of proper accommodation, reliable transportation, or threats to their personal safety and security [10]. As a result, providers may feel more entitled to engage in behaviors that result in high personal profit while reducing commitment to serving the needs of the community.

### Intrinsic motivational factors

In contrast to the factors described above, intrinsic motivation is grounded in the personal enjoyment and satisfaction of the individual provider rather than for the achievement of financial reward or other extrinsic incentives. Further, high levels of intrinsic motivation are linked to self-efficacy, with the result that healthcare providers will strive for high-quality service delivery even when the external environment is not ideal [11]. We suggest that providers with a strong sense of professional identity are less likely to engage in negative behaviors. We further suggest that changes to the admission process for medical and nursing schools in LMICs, along with some curriculum reform, can result in a workforce that is more intrinsically motivated. Although reform will require cooperation and consensus among ministry of health officials, and institutions of health professionals’ education, once in place, the financial and logistical resources required to impact intrinsic motivation are low relative to the cost of altering extrinsic factors.

## Standard protocols for medical school admission

**Fig. 1:**
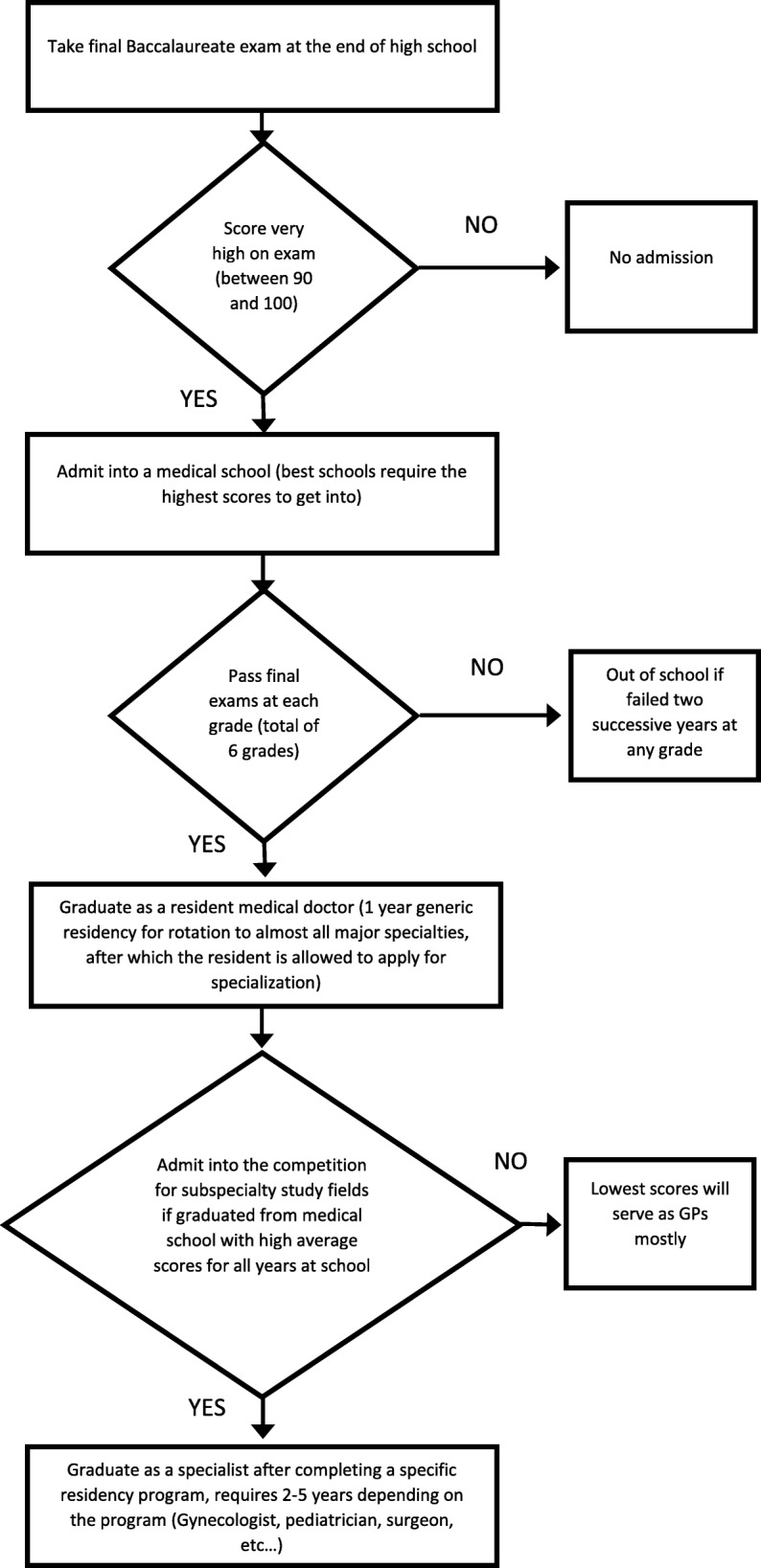
Current pathway to medical doctor degree

The current process for admitting students to medical and nursing schools in many LMICs is based on practices established during the colonial rule. This process is rigid, typically admitting only those students who score exceptionally well on exams taken at the end of secondary school (Fig. 1), although exceptions may be made for wealthy candidates. In many settings, secondary school students scoring less than 90% on their final exam are immediately ineligible for medical school. In Iraq and Nigeria, for example, students exiting secondary school must score above 90 on their final exam in order to be accepted to medical schools across the country [12]. While intellectual capacity for learning is one important component of competence, this process ignores other important qualities such as affinity for healthcare delivery and commitment to working in rural areas. As an example of an alternative model to this admissions process, a network of 13 medical schools (THEnet) in different parts of the world began recruiting medical students from underserved areas in 2008 and some of these schools are already able to demonstrate substantially greater medical coverage in rural areas [13–15]. Additionally, medical and nursing schools that participate in solicitation of sizable illegal or unfair payments to secure admission are more likely to exclude well-qualified students from lower socio-economic backgrounds or poorer regions of the country. This may result in the exclusion of students who are more intrinsically motivated to provide high-quality care, especially in rural areas. In India, for example, although exam requirements have been recently relaxed to allow entrance to lower-performing students, middle class or poor students were still excluded due to their inability to pay large capitation fees. This may explain, in part, why India has failed to see improvements to their healthcare system following implementation of a system originally designed to exclude wealthy but non-meritorious applicants.

Below we make recommendations designed to create a more flexible and inclusive admissions process, allow for alternate pathways into medical and nursing school, and promote curriculum that fosters not only technical competence but also those characteristics that support high-quality service delivery such as compassion, empathy, and altruism. We suggest the recommendations below will reduce the likelihood of intrinsically motivated candidates being excluded in favor of applicants who are wealthy but otherwise lacking qualification.

## Recommendations for increasing providers’ intrinsic motivation

### Reform admissions to increase likelihood of enrolling students with strong intrinsic motivation

**Fig. 2:**
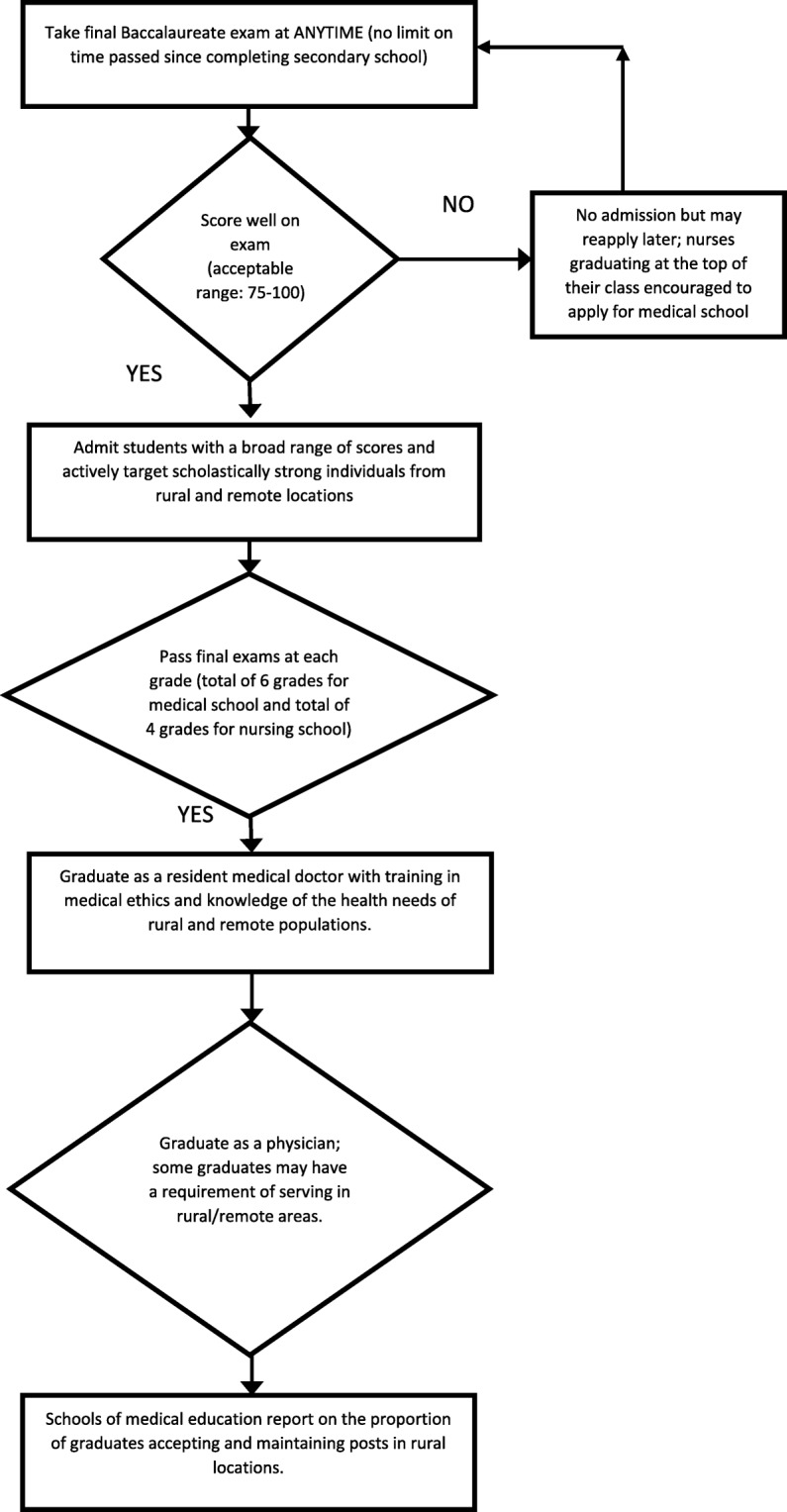
Alternative pathways to medical doctor degree

- Loosen rigid entrance exam requirements and admit students with a broader range of scores. Per the alternative admissions model implemented by medical schools in THEnet, include the background of the student (rural versus urban and family educational background) as elements considered in the admissions process.
- Actively seek and target scholastically strong individuals from rural and remote locations.
- Protect the number of admissions slots for government-sponsored students (relative to privately sponsored students) and also enforce consequences for individuals or institutions found to accept bribes in exchange for admission; this may reduce exclusion of middle class/poor and/or rural students.
- Create alternative pathways into medical school (Fig. 2). Many nurses and clinical officers complete a terminal medical degree in secondary school. Some of these clinicians may be interested in a career as a doctor and may already be working in rural areas. Allowing people to apply to medical school, regardless of the amount of time that has passed since they completed secondary school, increases the number of pathways to physician training.

### Expand curriculum to foster intrinsic motivation during training

- Expand medical curriculum to cultivate and foster a strong sense of professional identity and commitment to public service among students, per the training model implemented by THEnet. In the context of healthcare provision, the term “professional identity” encompasses the extent to which providers consider themselves part of a larger group of medical professionals; strongly identifying with the medical profession is likely to be predictive of an individual’s willingness to prioritize their duty to the patient’s well-being over their own personal gratification. Lack of professional identity contributes to the high prevalence of negative provider behaviors.
  - Create a cultural norm within medical education that places value on prioritizing the health needs of the local community over personal gains and rewards.
  - Implement learning objectives that emphasize the health needs of rural and remote areas.
  - Discourage styles of communication that contribute to a sense of superiority among providers and instead encourage language that highlights respectful and dignified treatment of patients.
- Develop metrics to measure the degree of professional identity among graduates of institutes of medical education and hold these institutions *accountable* for the performance of their graduates. Potential accountability measures include publishing medical education statistics such as:
  - The proportion of doctors and nurses who accept rural or isolated posts,
  - The proportion of doctors and nurses who stay in rural or isolated posts, and
  - The proportion of doctors and nurses who achieve high performance standards.
- Demonstrate how to give and receive feedback and implement a peer review system that can model how healthcare providers can support each other and behave as managers.

## Conclusion

In summary, negative provider behaviors are common within LMICs and result in harmful outcomes for both the patient and the provider. While extrinsic factors may foster and facilitate these negative practices, it is not often feasible to alter the external environment in low-resource settings. In contrast, healthcare professionals with strong intrinsic motivation and a desire to serve the needs of their community are less likely to engage in these negative behaviors and may draw upon internal incentives to deliver a high quality of care. Reforming medical education admission and training practices is one promising strategy for increasing the prevalence of medical professionals with strong intrinsic motivation.

## Footnotes

1 With the term “healthcare provider” we refer to both doctors and nurses.
